# Retention of the Urine, Caused by a Calculus in the Urethra, with Remarks as to Some of the More Prominent Causes of Vesical Retention in the Young

**Published:** 1862-05

**Authors:** Swayne Wickersham


ARTICLE XIV.

### RETENTION OF URINE,

### CAUSED BY A CALCULUS IN THE URETHRA, WITH REMARKS AS TO SOME OF THE MORE PROMINENT CAUSES OF VESICAL RETENTION IN THE YOUNG.

By SWAYNE WICKERSHAM, M.D.

Read before the Chicago Medical Society.

On the 18th of October, 1861, at 5 o'clock, P.M., I was called to South Dearborn Street, to see a child two years and nine months old. I was informed that forty-eight hours previously the boy had returned from a neighbor's, where he had been playing, apparently in good health and spirits. A few minutes after entering his parents' house, he uttered loud exclamations of pain and fell prostrate upon the floor. When he was resuscitated, he expressed a most urgent desire to micturate, but unable to void any urine. He was very restless throughout the night and suffered excruciating pain. The following morning, about seventeen hours after his first illness, a physician was summoned, who diagnosed suppression of the urine, and believed his case free from danger. He prescribed diuretics and antiphlogistics. The medicines were administered as ordered, until I saw him about thirty-one hours later. His symptoms were then significant of great danger. He was semi-comatose, could only be partially aroused, and if let alone his dulness would immediately return. His respiration was stertorous. His pulse were weak and frequent, face pale and heat diminished. His skin was heavily bedewed with a cold and clammy perspiration. His tongue was dry and brown.

His breath and the cutaneous exhalation emitted a urinous odor. He seemed to prefer to rest on his side, with the lower extremities drawn upwards. He had several serous alvine evacuations during his illness.

No urine had been voided although his desire to do so had been, until a few hours previously, most urgent. His abdomen was enormously distended, very much resembling an aggravated case of ascites, and the skin covering it presented that smooth, shiny appearance that is observed in that disease. The superficial veins were greatly enlarged and gorged with blood, but notwithstanding all this, I was enabled to detect a circumscribed tumor in the lower part of the abdomen, which I knew to be the bladder distended with the urine which had accumulated during the past fifty-five hours, not only from the natural secretion, but from kidneys stimulated by diuretics. Pressure upon the hypogastrium seemed to cause much pain. The perineum was tender and swollen. The tissues of the penis were tumefied and hardened, and the glans and prepuce to such a preternatural extent, as to cause phymosis. In order to ascertain if there was any urethral obstruction, I took a small instrument and when I had inserted it to the depth of half an inch from the *os urethrœ*, I felt it rest agains an impediment which gave the sensation characteristic of the presence of a metallic substance. The cause of the retention was now manifest. I thought it probable the boy, ignorant of the serious consequence which might result, had, while playing with his penis, as little boys are often observed to do, introduced the offending material into the urethra, a supposition that was incorrect. I had not with me forceps adapted for the removal of the calculus. I therefore endeavored to loosen it from its bed, with the small instrument already inserted. I then withdrew the instrument, and by making forward pressure along the urethra, in the rear of the location of the obstruction, and retracting the glans in a methodical manner, I soon brought into view the smallest extremity of the calculus. I then took a firm hold of the exposed portion, and maintaining the pressure to which I above alluded, although being compelled to exert considerable force, I was soon enabled to free the canal. A full stream of urine followed its removal. I did not measure the quantity, but full aware of the great power of expansibility possessed by this viscus, the amount, judging from the vigor of the stream, and the duration of the flow, would seem incredible. When the urine ceased to pass, gentle pressure above the pubis, caused a very great additional quantity to be voided.

The tumefaction of the abdomen immediately very much abated, but the muscles remained very flaccid. The little sufferer's constitutional symptoms were not improved. The condition of the respiration and circulation made me apprehensive that dissolution would soon ensue. I ordered that he should be immersed in mustard water, after which turpentine should be applied to the spine, sinipisms to the extremities, fomentations to the abdomen, gentle pressure over the bladder every hour, and that a drachm of brandy should be given every half hour. Deglutition was almost impossible, and the boy was bordering upon complete coma. He at intervals passed a considerable amount of urine, and especially when the pressure was made as above described. It was twelve hours before his grave symptoms commenced to perceptibly abate; but after this time, his improvement was rapid, and very soon a urinous odor could no longer be detected in his breath, or in the exhalation from the cutaneous surface. His respiration and pulse were almost normal, and a brief continuation of the stimulant completed the convalescence. The calculus that accompanies this paper shows that it was formed in the bladder. There can be no doubt but that it entered the urethra when he first manifested severe illness. It descended with the smallest extremity forwards. This was the position most favorable for its transmission. It is somewhat elongated. Its longest diameter is seven-twelfths of an inch, and its greatest circumference is five-sixths of an inch. It is the largest calculus that I have known to pass the urethra of a child, and it is to me marvelous that it did so successfully.

This case illustrates to us the necessity of a correct diagnosis, especially in cases of such gravity. Vesical retention should not be confounded with suppression of the urine. It is an inexcusable error to do so. Yet there are complications where, unless a physician be upon the alert, he may commit a lamentable mistake. In a very fat individual, or one with a tympanitic abdomen, it will not answer to rely upon the touch. Percussion, in this case, will furnish a better guide, the part occupied by the distended viscus will yield a less resonant sound. It is truly astonishing to what an extent the bladder will sometimes expand, before there will be a destruction of any part of its parietes. Cases are on record where it has reached the scrobiculus cordis, and frequently the fundus has been as high as the umbilicus. Cases are mentioned where sixteen pints of urine have been evacuated from the bladder of an adult, and five and six quarts are not very unusual.

MACKINTOSH mentions a case of vesical retention, where the fundus of the bladder became attached to the tissues in the vicinity of the umbilicus, and from which the urine subsequently escaped. BILLARD says he has found it in children to fill the whole cavity of the abdomen; and HOWSHIP and PARRISH have found it under such circumstances to contain twenty ounces, and the ureters to acquire a diameter of an inch or more. Much greater quantities are mentioned by others. It has been mistaken for ascites, when distended so as to occupy almost the entire abdominal space.

Such cases have been mistaken by some of our great men. Prof. GIBSON, of Philadelphia, was called to a child about two years of age, supposed to labor under ascites, and so strongly did it resemble the disease, that he at first took it to be a case of the kind, but upon learning the history of the case he was induced to examine the urethra, where he found a calculus. Upon enlarging the orifice with a lancet, the stone was instantly pushed out, and followed, to his surprise, and that of a medical attendant, by two quarts of urine. Dr. PARRISH reports a similar case. Sir EDWARD HOME relates an instance in which the celebrated Mr. HUNTER actually tapped the bladder, supposing the case to be one of abdominal dropsy.

But such cases are anomalous. Usually before it will submit to such an expansion, there will be sloughing or ulceration of some part of its parieties or that of the urethra, and the urine will escape into the tissues adjacent to the rupture. In such cases, or those complicated with ascites, in addition to other means, the introduction of a finger into the rectum, or a catheter into the bladder will be sufficiently diagnostic. When the retention is partial and the accumulation takes place gradually, the viscus accommodates itself, to a certain extent, to the additional pressure, and great distention may occur without the patient suffering much pain; but unless discovered and relieved the same symptoms will occur as in the acute form, and the same fatal termination. I have reason to believe that this form of retention is often overlooked. In diseases attended with great prostration, where there is deficient innervation, and patients are only partially conscious, as is often observed in typhoid fever, I have known it to be disregarded. The attendants may think that sufficient urine has been voided. It may pass involuntarily, the bladder having lost to a degree its contractile power, and yet the system is being prejudicially influenced from urinary retention. I am well satisfied that the catheter is not used in these cases as frequently as it should be.

The retention of urine in new-born infants is often overlooked or carelessly regarded as suppression. The obstetrician sometimes prescribes, at his office, diuretics for such cases. The symptoms are aggravated. He now goes to see the infant, but irremediable damage has been done. I have known an instance of the kind. In such cases, the obstruction will, in most instances, be found at the neck of the bladder or in the urethra, and I believe almost always in the latter. The retention may be complete or partial. It may originate from inspissated mucus. This cause by many is regarded as very frequent. CONDIE thinks it very rare, having met with but two cases in thirty-six years' practice as an obstetrician. Perhaps a more common cause is an occlusion of the urethra by a thin membrane, situated at or near the orifices. Adhesion of the lips at the external orifice, sometimes impedes the flow. Morbid irritability at the neck, and inflammation may be enumerated as causes. Spasmodic contraction at the bulb of the urethra is sometimes assigned as a cause. I think the last three causes to be very rare in the new-born. The obstruction is seldom permanent, but almost always remediable, and requires but the medical attendant to have a correct comprehension of the difficulty.

In such cases, even though the impediment is not removed, rupture of the bladder does not seem to be very frequent, but the infant mostly perishes from paritonitis or cephalic disease. The cases of partial retention in infants are the more fruitful source of error. Congenital or induced phymosis has latterly been added to the causes of more or less permanent retention of the urine. This cause is now attracting considerable attention. Sir CHARLES BELL, Mr. GUTHRIE, and other well known writers on diseases of the urinary organs, have not alluded to it as a cause of urethral and vesical affections in the young.

Mr. PRICE recently, in a very able paper, read before the London Medical Society, stated this to be one of the most common deviations from the normal construction of the preputial and glandular portions of the penis; and stated his conviction that it was one of the most frequent causes of genito-urinary disturbance. He cited a series of cases of retention which originated from this abnormal condition, and urged that it should be promptly corrected. There are other causes of the trouble. The subject is full of interest, not only vesical but renal retention. The physician, in this as well as in many other diseases, gives too much credence to the statements of attendants, and is thus led to form, in some cases, a very erroneous and fatal diagnosis.

Cases of rupture of the bladder are reported, where the infant's sufferings were confidently attributed to other causes. Hence the necessity of a personal examination, in order that his appliances may be the correct ones. A physician cannot be free from censure, if he has the medical care of children with symptoms indicative of urinary retention, if he neglects to institute a cautious, careful, and thorough examination, that will reveal the true condition, notwithstanding the assurances of the ordinary attendants, that no difficulty need be apprehended.

My remarks have been intended to apply chiefly to the young. Not to discuss the treatment of the disease, but to glance at some of the more prominent causes of vesical retention. It would require many additional pages to discuss this subject in the full and systematic manner it deserves. There are few diseases more fruitful in evil and error, and certainly merits more consideration than is usually allotted to it.

